# Comparison of reproductive performance of AI- and natural service-sired beef females under commercial management

**DOI:** 10.1093/tas/txab114

**Published:** 2021-06-30

**Authors:** Mackenzie A Marrella, Robin R White, Nicholas W Dias, Claire Timlin, Stefania Pancini, J Currin, Sherrie Clark, Jamie L Stewart, Vitor R G Mercadante, Heather L Bradford

**Affiliations:** 1 Animal and Poultry Sciences, Virginia Tech, Blacksburg, VA 24061, USA; 2 Virginia–Maryland Regional College of Veterinary Medicine, Virginia Tech, Blacksburg, VA 24061, USA

**Keywords:** age, age at first calving, body condition score, calving interval, cattle, pregnancy

## Abstract

The objective of this study was to assess differences in reproductive performance of natural service and artificial insemination (**AI**) sired beef females based on pregnancy outcomes, age at first calving, and calving interval. Data were sourced from 8,938 cows sired by AI bulls and 3,320 cows sired by natural service bulls between 2010 and 2017. All cows were in a commercial Angus herd with 17 management units located throughout Virginia and represented spring and fall calving seasons. All calves were born to dams managed with estrus synchronization. Pregnancy was analyzed with generalized linear mixed models and other reproductive measures with linear mixed models in R. Six models were evaluated with the dependent variables of pregnancy status at the first diagnosis, pregnancy status at the second diagnosis, pregnancy type (AI or natural service) at the first diagnosis, pregnancy type at the second diagnosis, calving interval, and age at first calving. Independent variables differed by model but included sire type of the female (AI or natural service), prebreeding measures of age, weight, and body condition score, postpartum interval, sex of the calf nursing the cow, and management group. No differences were observed between AI- and natural service-sired females based on pregnancy status at first and second pregnancy diagnosis (*P* > 0.05). Sire type was only found to be significant for age at first calving (*P* < 0.05) with AI-sired females being 26.6 ± 1.6 d older at their first calving, which was expected because AI-sired females were born early in the calving season making them older at breeding. Surprisingly, age and body condition score were not significant predictors of pregnancy (*P* > 0.05). Body weight at breeding was not significant for pregnancy (*P* > 0.05) but was significant for age at first calving (*P* < 0.05). These data suggested that lighter heifers calved earlier which contradicts our original hypothesis. Overall, commercial Angus females sired by AI or natural service bulls had similar reproductive performance. Factors that were commonly associated with reproductive success were not significant in this commercial Angus herd managed with estrus synchronization. Given the size of these data, the importance of body condition, age, and weight should be reassessed in modern genetics and management practices.

## INTRODUCTION

Artificial insemination (**AI**) as a technology has several major benefits. One major advantage is the ability to use semen from genetically superior bulls, enabling producers to make more precise mating decisions by mating each cow to a specific bull. Another major advantage to using AI is the ability to reduce the number of bulls that are being kept on the farm making it safer for workers (Brackett et al., 1981) and reducing bull maintenance costs, potentially increasing profitability. Based on these advantages, improved adoption of AI would be beneficial for the beef industry. According to the latest USDA (2020) data, only 11.6% of beef cows are bred using AI compared with the dairy industry, where 89% of operations use AI (USDA, 2018). Beef producers do not adopt AI practices primarily because of the time and labor required. Other top reasons included inadequate conception percentages, lack of facilities, cost, and the complexity of the technology (Whittier, 2010).

Reproductive outcomes are complex with many factors involved, and one important factor is nutrition. Sufficient energy and appropriately balanced sources of amino acids, vitamins, and minerals are needed for cows to begin cycling and exhibiting estrus (Rickards et al., 1986; Randel, 1990; Hess et al., 2005). If there is a delay in exhibiting estrous, the conception percentage will be negatively affected during that breeding season. Another important factor is time of calving. Cows that were able to cycle several times before the breeding season were more fertile than cows that were bred on their first cycle after calving (Nelson and Beavers, 1982). Wiltbank et al. (1962) found cows that were fed high-energy diets, especially postcalving, showed greater pregnancy percentages. Greater levels of precalving nutrition were associated with shortened postpartum anestrous periods (Wiltbank et al., 1962). Season and environmental factors also play a role in pregnancy percentage. During very hot weather, the animal experiences heat stress, which causes a significant reduction in conception percentages (Dunlap and Vincent, 1971). Lastly, age of the female has an effect on pregnancy percentage as well as the calving interval. Calving interval is the period of time between two consecutive calvings in the same female and is often used as a measure of reproductive efficiency. Other research has found that 2-yr-old cows and old cows tend to have lesser conception percentages than cows in the middle age group (Short et al., 1990; Osoro and Wright, 1992). First calf heifers often experience a longer calving interval and later calving dates than mature cows (MacGregor and Casey, 1999) because of greater energy requirements after their first calving. The heifers need to put more energy toward their growth and maintenance than the mature cows; so, heifers have less energy to put toward reproductive functions (DeRouen et al., 1994). Improving our understanding of how these different factors interact with breeding approaches may help to better define situations in which AI will be successful for commercial beef operators.

Much of the research on factors related to pregnancy outcomes is decades old and may not still be applicable to modern beef cattle genetics as cattle today are generally lighter at birth, heavier at all other ages, more muscular, and produce more milk (AAA, 2021). The objective of this study was to quantitatively characterize how prebreeding measures of body condition score, weight, age, and sire type (AI or natural service) affected pregnancy outcomes in a large, commercial beef herd. The hypothesis was that females with the greatest reproductive outcome would be sired by AI, have greater body condition scores, be heavier, and be older.

## MATERIALS AND METHODS

Animal Care and Use Committee approval was not needed as data were obtained from preexisting databases and were used for a retrospective observational study. Reproductive data were obtained from the Virginia Department of Corrections beef cattle herd. Cattle were housed in 17 locations throughout Virginia and consisted of spring and fall calving herds. Data were available from spring 2010 to spring 2017 with the exception of spring 2011. Descriptive statistics are presented in Table 1. The mean (SD) number of cows per location-year-season was 88 (47). The cattle were typically Angus and Angus-influenced, but records were not detailed enough to further evaluate breed composition. The AI sires were selected through a long-term contract with a bull stud and were individually mated to cows based on known lineage. Natural service sires were leased from an Angus breeder and were used for 2 yr. All natural service bulls passed a breeding soundness exam 60 d before the breeding season. Cows and heifers were estrus synchronized using common industry protocols that varied from year to year. Generally, controlled internal drug release (CIDR) were used in conjunction with a CO-Synch or Ov-Synch protocols for 5 or 7 d. All animals were bred by AI, and bulls were turned out 10 d after synchronization for approximately 70 d. Prior research indicated a sensitivity of 0.58 for an activated estrus detection patch and a confirmed AI pregnancy (Mercadante et al., 2019). During synchronization, individual body weight and body condition scores were assessed. Pregnancy diagnosis was performed through rectal ultrasonography at approximately 55 to 65 d after AI and again between 35 and 45 d after the breeding season. Fetal ages were used to determine whether the cow was pregnant by AI or natural service, and ages were corroborated with calving dates when available. Sire information was tracked by the farms when known and was reconciled with the dam’s breeding record and calving date when available. Animals with sire inconsistencies were removed (*n* = 122) from the database prior to analysis, resulting in a data set of 8,938 cows sired by AI bulls and 3,320 cows sired by natural service bulls.

**Table 1.** Descriptive statistics for age, days postpartum, body condition score, and weight in commercial Angus cows in Virginia

Variable	Minimum	Mean	Maximum	SD
Age at first calving, d	641	728	465	22
Calving interval, d	304	369	465	22
Days postpartum, d	12	79	148	18
Scaled days postpartum^1^	−3.6	0.0	3.8	1.0
Weight, kg	291	551	857	85
Scaled weight^1^	−6.3	0.0	4.0	1.0

^1^Weight and days postpartum were rescaled with a mean of 0 and a variance of 1.

### Statistical Analysis

Pregnancy data were analyzed with a generalized linear mixed model. Continuous data were analyzed with linear mixed models. Details of those models are described below. Interactions between sire type and other fixed effects were tested, but Akaike’s information criteria (**AIC**) and Bayesian information criteria (**BIC**) statistics favored models without interactions. Data were analyzed in R using the lme4 package (Bates et al., 2015). The DHARMA package (Hartig, 2019) was used for residual diagnostics for the generalized linear mixed models, and the emmeans package (Lenth, 2019) was used for contrasts.

### Pregnancy

**Figure 1.**
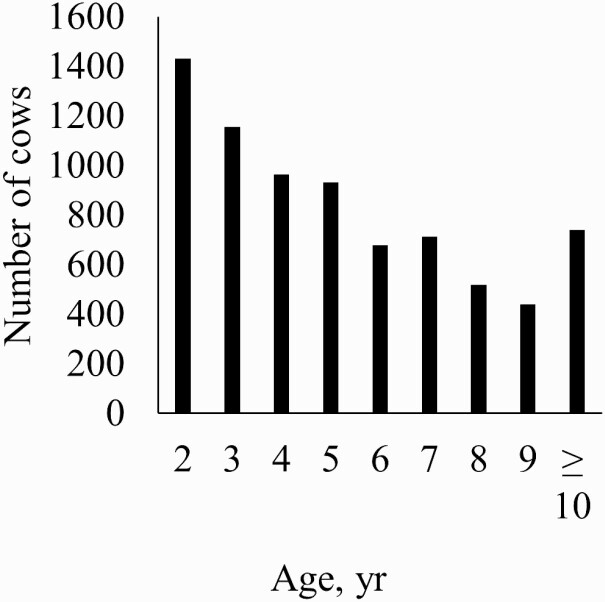
Histogram of the number of cows in each age group for a commercial Angus herd in Virginia.

**Figure 2.**
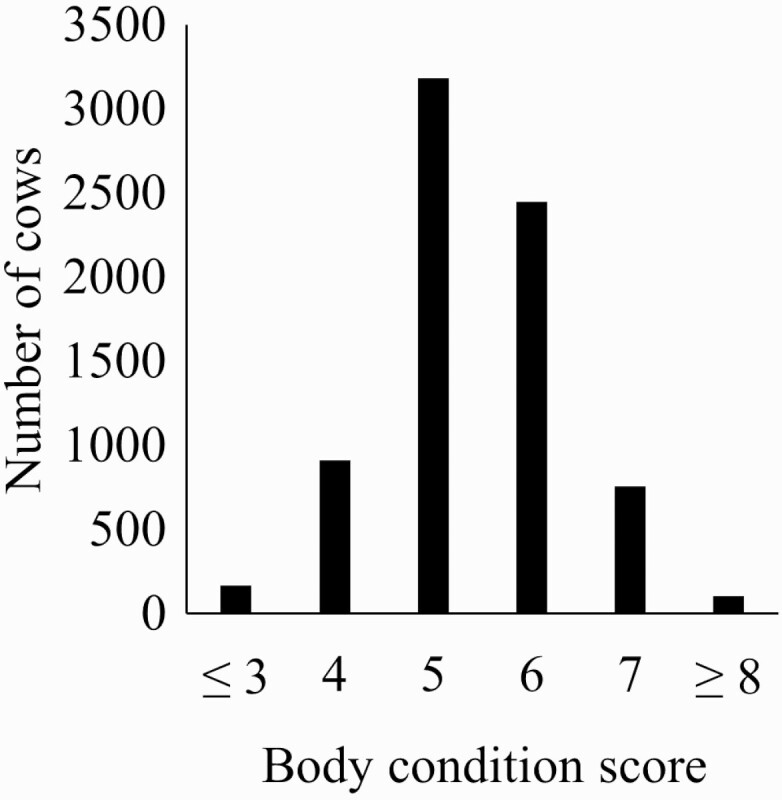
Histogram of the number of cows based on body condition score for a commercial Angus herd in Virginia.


A subset of data was used with complete records for all variables included in a given model. Ages were grouped by year with the exception of the oldest group being 10 yr or older (Figure 1). Ages were categorized by year as is common in Beef Improvement Federation (2021) recommendations and to avoid assumptions of linearity. Body condition scores were grouped into categories of less than or equal to 3, 4, 5, 6, 7, and greater than or equal to 8 (Figure 2) because of few records at the extreme values. Records were removed for cows with weights less than 227 or greater than 907 kg. Weight and days postpartum were rescaled to have a mean of 0 and a variance of 1 for model stability. Breeding treatment (generally synchronization protocol) was tested as an additional grouping variable, but the models did not converge. Data (*n* = 5,966) were analyzed with the following repeated records model separately for the first and second pregnancy diagnosis:

$$y_{ijklm}=sire_{i}+calfsex_{j}+age_{k}+\beta_{1}days_{n}+\beta_{2}weight_{n}+cg_{l}+animal_{m}+e_{ijklmn},$$

where *y* was a binary pregnancy observation at either the first or second pregnancy diagnosis, *sire* was a class variable indicating if the female’s sire was an AI or natural service sire, *calfsex* was a class variable for the sex of the calf nursing the cow at the time of breeding (heifer or bull), *age* was a class variable for the age category at breeding, *β* were fixed regression coefficients, *days* was a covariate for the scaled number of days postpartum at AI breeding, *weight* was the cow’s scaled weight at AI breeding, *cg* was the random contemporary group (location–year–season), *animal* was the random animal effect for repeated observations on the same female, and *e* was the random residual. All random effects were independent and normally distributed with 0 mean and homogeneous variance.

In addition, differences were evaluated in pregnancies resulting from AI or natural service matings (pregnancy types at first and second pregnancy diagnosis). Data were further edited to include only cows who were pregnant at the first pregnancy diagnosis (*n* = 4,174) and separately at the second pregnancy diagnosis (*n* = 4,087). Data were analyzed with the same model described previously, but *y* described if the pregnancy resulted from an AI or natural service mating.

### Calving Interval

Calving interval was calculated as the number of days between consecutive calving events. Data were then edited to include only animals with a calving interval (i.e., animals retained in a herd for successive breeding seasons). This restriction resulted in an average of 4.4 observations on 2,122 cows. Calving interval observations were limited because of missing data in spring 2011. Data were analyzed with the following mixed model:


$$y_{ijklm}=sire_{i}+age_{j}+\beta_{1}weight_{n}+BCS_{k}+cg_{l}+animal_{m}+e_{ijklmn},$$

where *y* was the calving interval, *weight* was the cow’s weight at the time of breeding, and all other effects were the same as previously described. A quadratic effect for weight was tested but did not improve model fit based on AIC and BIC statistics.

### Age at First Calving

Age at first calving in days was calculated for all animals with a known birth date. Data were edited to include only heifers that calved by 800 d of age, eliminating heifers that did not conceive in the first breeding season and were held over for the following season. This resulted in 751 records for analysis. Data were analyzed with the following mixed model:


$$y_{ijk}=sire_{i}+\beta_{1}weight_{j}+cg_{k}+e_{ijk}$$

where *y* was the age at first calving and all other effects were the same as previously described. A quadratic effect for weight was tested but did not improve model fit based on AIC and BIC statistics.

## RESULTS AND DISCUSSION

### Sire Type

The sire type was not significant for any pregnancy analyses or calving interval (*P* > 0.05) but was significant for age at first calving (*P* < 0.01, Table 2). Females sired by AI were 26.6 ± 1.6 d older at their first calving compared with natural service-sired females. The dams of these females were synchronized for AI breeding resulting in AI-sired calves being born earlier in the calving season. The AI-sired heifer calves were at least 21 d older than natural service-sired heifer calves if all the cows responded to the estrus synchronization protocol. Once these heifer calves were old enough to breed, both the AI and natural service-sired females were bred at the same time. Hence, AI-sired heifers were older at first breeding and first calving because the heifers were initially born at the beginning of the calving season and were older than their natural service-sired contemporaries. Despite being older at the time of breeding, no other reproductive differences occurred based on the female’s sire type. This result could be because expected progeny differences for reproductive traits are relatively recent. Producers have not had as much time to select for and to improve these traits, suggesting there might not be as large of differences between AI and natural service bulls for reproductive traits. For example, the Angus breed has genetically increased the pregnancy percentage of a sire’s daughters by about 1% over the past 30 yr (AAA, 2021). Unfortunately, expected progeny differences were not recorded for all bulls at the time of selection, and some natural service bulls were not registered nor tracked beyond a tattoo number resulting in no available genetic information. The authors are unaware of other research comparing reproductive function of AI- and natural service-sired females.

**Table 2.** *P*-values for effects in final models evaluating pregnancy status at the first and second diagnosis, type of pregnancy (artificial insemination- or natural service-sired) at the first and second diagnosis, calving interval, and age at first calving in commercial Angus cows

Model	Sire type^1^	Weight	Age	Calf sex	BCS	Days postpartum
Pregnancy diagnosis 1	0.81	0.09	0.20	0.02	0.22	0.40
Pregnancy diagnosis 2	0.70	0.99	<0.01	0.80	<0.01	0.40
Mating type diagnosis 1	0.77	0.19	0.22	0.02	0.39	0.49
Mating type diagnosis 2	0.80	0.09	0.20	0.02	0.22	0.40
Calving interval	0.16	0.17	<0.01	0.24		
Age at first calving	<0.01	<0.01				

^1^Sire type indicated if the female’s sire was an artificial insemination or natural service bull.

### Age

**Figure 3.**
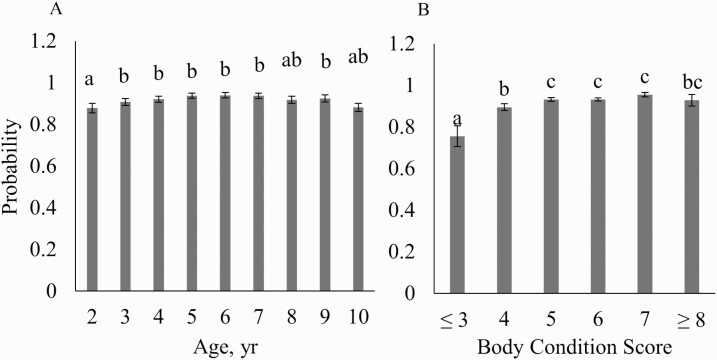
Mean pregnancy probabilities (SE) during the first pregnancy diagnosis based on age and body condition score in commercial Angus cows in Virginia.

**Figure 4.**
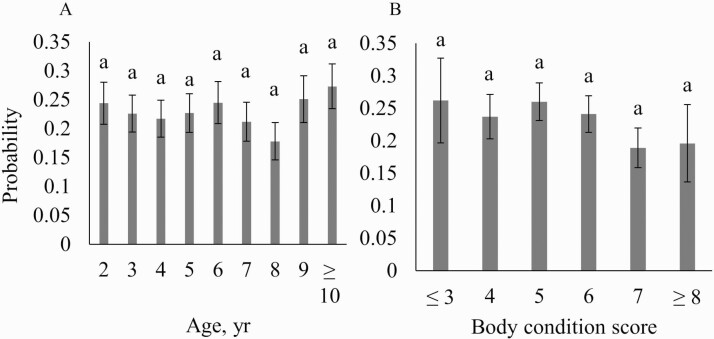
Mean pregnancy probabilities (SE) during the second pregnancy diagnosis based on age and body condition score in commercial Angus cows in Virginia.

Age was not significant for any pregnancy variables (*P* > 0.05) but was significant for calving interval (*P* < 0.01, Table 2). When compared with the older cows, 2-yr-old females had lesser odds of being pregnant by the first pregnancy diagnosis (Figure 3) but were no different from the mature females at the second pregnancy diagnosis (Figure 4). These results indicate that the 2-yr-old females were slower to rebreed than the older cows but had no difference in the final pregnancy outcome. The 2-yr-old females had a slightly lesser average body condition score (0.4 units) than mature cows, which could contribute to delayed pregnancy. A study by Goehring et al. (1987) found that a 2-yr-old female needed a calving body condition score of 6 to successfully rebreed. Our 2-yr-old females had an average body condition score greater than 5 at breeding indicating that the females were well managed.

**Figure 5.**
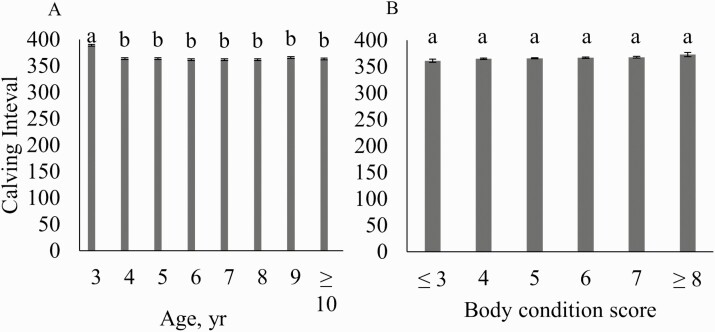
Least squared means (SE) for calving interval based on age and body condition score for a commercial Angus herd in Virginia.

The 3-yr-old females had a significantly longer calving interval than females at other ages (Figure 5). Heifers were bred to start calving about 10 d before the cowherd and were then bred with the rest of the cows to have their second calf. This management practice gives the 2-yr-old females an extra 10 d before being bred. The longer calving interval for 3-yr-old females was a combination of management and being slower to rebreed as 2-yr-old females because the difference was generally around 25 d (Figure 3). These cows would have been going through the nutritional challenge of their first calving and their first lactation. Young cows would also be growing at this time which means these females would be putting more of their energy into growth and less into reproductive functions (DeRouen et al., 1994). The combined effects of both of these factors could lead to delays in estrus, conception, and calving, which could explain why there was a longer calving interval in the 3-yr-old cows (Rae et al., 1993).

### Body Condition Score

Body condition score was not found to be significant in any of the models (*P* > 0.05, Table 2). However, in the model evaluating pregnancy percentage at the second pregnancy diagnosis, the cows with body condition scores of 3 and 4 had lesser odds of being pregnant when compared with the greater body condition scores (Figure 3). The cows with a lesser body condition score have less body reserves available to put towards becoming pregnant. The cow’s energy reserves are used for maintenance first, then whatever is left is used for production. In the thinner cows (body condition score [BCS] ≤ 4), most of the net energy will be used for maintenance functions rather than for reproduction. Body condition score was also negatively correlated with postpartum interval; so, as body condition score decreases, postpartum interval increases. Houghton et al. (1990) showed that postpartum intervals were extended in thinner cows to over 80 d. Their results showed that in order to maintain an acceptable postpartum interval, cows need to maintain a body condition score of 5−6. Despite a documented relationship between body condition score and rebreeding, no significant differences existed for calving interval in these data (Figure 5). These cows were well managed with most having a body condition score of 5 or 6 with relatively few extremes, which could contribute to the lack of observable differences. In addition, the use of progesterone-based estrus synchronization protocols will aid females in reproductive performance as these protocols often induce cyclicity. This effect could explain the lack of any inherent differences in calving intervals based on body condition score.

### Additional Variables

The sex of the calf nursing the cow was a significant predictor (*P* < 0.05) for pregnancy type at the first and second pregnancy diagnosis. The odds ratio (SE) for pregnancy with a bull compared with heifer was 0.96 (0.06) but was not significant (*P* = 0.50). Calf sex was a significant predictor in the model, but the odds ratio comparing the two sexes was not significant. However, calf sex was not a significant predictor for calving interval (*P* > 0.05). Raising one sex over the other was not associated with the dam’s subsequent calving interval despite bull calves typically being larger. Even though it was not considered in this study, one other thing to consider is the sex of the fetus. The sex of the fetus was expected to be a significant predictor of calving interval based on previous research done by Nogalski and Piwczyński (2012) in dairy cattle. The authors found that male fetuses tended to have a gestation length that was 1.8 d longer than female fetuses.

Weight was found to be significant for age at first calving (*P* < 0.01) but was not significant for pregnancy type at first and second pregnancy diagnosis, and calving interval (*P* > 0.05, Table 2). For age at first calving, the regression coefficient (SE) for weight was 0.014 (0.005) kg/d. These results contradict with our original hypothesis and with the literature. A study performed by Boligon and Albuquerque (2011) showed that heavier heifers calve earlier when compared with lighter heifers. Weight was expected to be a significant predictor of pregnancy based on previous research done by Selk et al. (1988) but was not in these data. Since that study was published, the average Angus cow is approximately 80 kg heavier based on the genetic trend for mature weight (AAA, 2021), which could contribute to weight differences being less important. Selk et al. (1988) found that cows whose body weight was maintained until calving had a greater pregnancy percentage than those who lost or gained body weight. Only weight at breeding was modeled, but the statistical models were more complex with contemporary groups as random effects to increase generalizability. Either of these could contribute to weight not being a significant predictor of pregnancy outcome. For the calving interval model, the regression coefficient for weight (SE) was 0.002 (0.002) kg/d but was neither significant nor practically important.

## CONCLUSIONS

When the AI- and natural service-sired females were compared based on pregnancy status by the first and second pregnancy diagnosis, there were no statistical differences between the two. Artificial insemination sires are typically genetically superior to natural service bulls for commonly measured traits like growth and carcass composition. Heifer pregnancy and fertility EPDs are relatively newer traits and lowly heritable; so, when these heifers were born, there would not have been a strong selection for those traits. Consequently, the AI bulls might not be as genetically superior to the natural service bulls for fertility traits. Further research needs to be done to determine whether other performance measures differ between AI- and natural service-sired offspring. Interestingly, age, body condition score, and weight were not significantly associated with pregnancy outcomes despite well documented, historical literature on these parameters. Data were from well-managed farms with good nutritional and reproductive management including the use of estrus synchronization programs, which could be altering relationships between reproductive performance and other factors that are typically predictive of successful reproductive outcomes. Additional research may be needed to better understand how estrus synchronization programs have affected the biology and management of beef cows.
